# Monthly Summary

**Published:** 1874-12

**Authors:** 


### MONTHLY SUMMARY.

*Local Use of Chloral.*—In the Atlanta *Med and Surg. Journal*, Dr. E. M. Nolan gives this case :—

"A few months ago a lady at her catamenial period requested relief from a very distressing and painful headache. I dissolved fifteen or twenty grains of chloral in very little water, and with the tip of one finger rubbed it upon one of her temples until she could very sensibly feel the burning, and until the skin was reddened. The part rubbed was not larger than a silver dollar, but when I ceased the pain was entirely relieved and remained so. I have used it in this manner many times since, in ordinary nervous headache, and have never been disappointed, save one time, and then the patient was partially relieved. I sometimes rub one temple, and sometimes both, according to the effect desired. There is no inconvenience from the application. The surface turns slightly red, and feels a little sore upon being touched, for two or three days. There is a slight desquamation of the epidermis, and soon there is no sign left of the application."

---

*Cutting Teeth.*—It was a peculiarity of this baby to be always cutting teeth. Whether they never came, or whether they came and went away again, is not in evidence ; but it had certainly cut enough, on the showing of Mrs. Tetterby, to make a handsome dental provision for the sign of the Bull and Mouth. All sorts of objects were impressed for the rubbing of its gums, nothwithstanding that it always carried, dangling at its waist (which was immediately under its chin,) a bone ring, large enough to have represented the rosary of a large nun. Knife-handles, umbrella-tops, the heads of walking sticks selected from the stock, the fingers of the family in general, but especially of Johnny, nutmeg-grater, crusts, the handles of doors, and the cool knobs on the tops of pokers, were among the commonest instruments indiscriminately applied for this baby's relief. The amount of electricity that must have been rubbed out of its gums in a week, is not to be calculated. Still Mrs. Tetterby always said "it was coming through, and then the child would be herself," and still it never did come through, so the child continued to be somebody else.—*Christmas Stories.*

*Lead as a Constituent of Enamel the Cause of Chronic Poisoning.*—The patient, a corpulent man, 36 years of age, suffered at one time from a severe attack of typhlitis. From this, however, he had recovered some time, when he began to complain of intense pain in the abdomen, and was considerably emaciated in a short time. His face became yellow and he continued to suffer from fulness and pain over the cæcal region, and shooting pains through the breast and stomach. Percussion over the abdomen was dull, sleep and appetite disturbed; pulse and temperature were normal. At first typhlitis was suspected, and a dose of castor-oil administered, after which a passage was obtained. The fæces consisted of large boggy masses. The stomach became reduced in size, but the pain still continued, extending over the back and through the extremities. The stomach was tense and contracted, and on applying pressure the pain was *not* increased but vomiting was produced almost instantly, consisting of green masses. The bowels were very constipated, and only moved when a drastic cathartic was administered. After four weeks had elapsed, a slate-colored band made its appearance on the gums, and bladder-tenesmus became apparent Upon this appearance a diagnosis of lead poisoning was arrived at. Soon after this the sister of the patient was similarly attacked.

The source of the lead poisoning was shortly afterward detected. The jar used as a receptacle for vinegar was found to be much corroded, and nearly all the enamel was removed by the vinegar. The vinegar possessed disagreeable sweetish taste. One "schoppen" was placed in the jar for ten hours, when on examination 0.09 grm. of metallic lead were found. The jar, when full of vinegar, must have contained, after standing ten hours, 1.8 grm., and after standing one week must have contained 30.24 grms. of lead in solution. About one jar of vinegar was consumed per day, which was quite sufficient to produce lead poisoning in the family.—*Med. Record.*

---

*How to Insert the Hypodermic Syringe.*—Prof. Hildebrandt, of Königsberg, says in the *American Journal of Obstetrics*:—

In performing the injection I take up a firm fold of skin and insert the canula of the syringe perpendicularly (not obliquely) into the crest of the fold, to the depth of one-half the length of the canula, in order that the fluid may always enter the thick, adipose, subcutaneous cellular tissue. I believe that it is owing to this circumstance alone that I have never had occasion to see an abscess. As a rule, only the first three to five injections are painful: subsequently they are more and more easily borne. A slight subcutaneous infiltration (*hautknoten*) always remains, in some women only several days, in others many days, and even weeks.—*Med. and Surg. Reporter.*

*Arsenic Poisoning by a Green Carpet.*—At a recent meeting of the Swedish Medical Society of Stockholm, Dr. Kjellberg related the case of a young man, who, having manifested symptoms of arsenic poisoning, was sent away to travel. During the following year, he enjoyed perfect health, but having at length returned home, he began to suffer, shortly after, in the same manner as before. Suspicion was now directed to a green carpet upon the floor of his chamber and an analysis revealed the fact that there was contained in the coloring matter a very considerable quantity of arsenic. The removal of this carpet was followed by an immediate disappearance of all the morbid symptoms.—*Hygiea, Stockholm.*

---

*How to Deprive Iodine of its Stain.*—Add a few drops of phenic acid to the tincture, and it will not stain ; moreover, the tincture is more efficacious, and its action more certain. M. Bogs recommends the following formula for use in injections ; Alcoholic tincture of iodine, three grm ; phenic acid, six drops ; glycerine, 30 grm ; distilled water, 150 grm. This preparation is superior to all others in the treatment of blennorrhagia and leucorrhœa,—*Med. and Surg. Reporter.*

---

*The Prevention of Dental Caries.*—Dr. Flagg, in the *Dental Cosmos*, states that he has found repeatedly the most beneficial effects produced by the administration of medicines which, used locally in the seemingly accepted method of experiment, would be disastrous in the extreme ; for example nitro-muriatic acid will be recognized as eminently destructive of tooth-tissue. He has not seen a case of dental carfes which he could attribute to the use of any acid medicine, while he has again and again seen remarkably prompt cessation of dental tenderness and tendency to caries, resulting from local weakness of tooth-structure consequent upon long-continued biliary difficulty, from the administration of fifteen to twenty drops of nitro-muriatic acid daily.

---

*Monobromide of Camphor.*—Dr. Bournville, of the Paris School of Medicine, strongly advocates the therapeutical employment of this compound in cases of delirium tremens, epilepsy, hysteria, infantile, convulsions (due to the irritation of teething,) chorea, paralysis agitans, etc. He gives the results of numerous interesting experiments, and also the histories of various cases treated successfully. In hydrophobia tetanus, and epilepsy, he recommends a solution of the monobromide in alcohol and glycerine to be injected under the skin.

The formula for this substance is $C^{10} HO^{16}$ Br.—*Med. and Surg. Reporter.*

*To Suppress Hemorrhage in the Mouth during Operations.*—Professor Andrews, of Chicago, (*Med Examiner,*) advises, when hemorrhage interferes with operations in the mouth, to use the atomizer with ether, after etherization by inhalation if the latter should be deemed necessary. He says that it does not interfere with the operation, that it restrains bleeding, and that the inhalation of the vapor from the spray keeps up a sufficient degree of anæsthesia.

---

*Electricity in Vomiting.*—This method succeeded admirably in controlling vomiting in the practice of Bellevue Hospital (*Med. Record*) In the vomiting of puerperal fever it had answered well. The positive pole is placed over the pit of the stomach. The strength of the current is varied according to circumstances.—*Pacific Med. & Surg. Journal.*

---

*Death from Chloroform.*—At Boston, on Saturday, October 3d, in the case of Linscott, who died in a dentist's chair from the inhalation of chloroform, the coroner's jury declared themselves of the opinion that, with our present knowledge of chloroform, its use as an anæsthetic is wholly unjustifiable, and they recommended the passage of a law forbidding its administration.—*Med. & Surg. Reporter.*

---

*Physical Peculiarities of Negroes.*—Dr. A. W. McDowell publishes, in the *American Practitioner*, some observations on this subject which contain some facts that are new to us. The negro's want of power of resisting disease was abundantly shown in the late war. Dr. McDowell states that the fine chests frequently seen among the males are due solely to the great development of the pectoral muscles, and that the lungs are decidedly less in weight than those of white men. The liver on the other hand is larger. He goes on to say, "The negro's lower bowel was smaller. The colored troops were much troubled with constipation, often requiring purgatives, while at the same time and place the white troops had diarrhœa. The most marked difference existed between the spleen of the black and that of the white, the former only weighing half as much as the latter. 'Ague cake' was one of the sequelæ of malarial disease observed among the whites, but not among the blacks." In his army practice, the author weighed the brain at every *post mortem*, and found that its weight increased in direct ratio to the admixture of Caucasian blood.

*To Arrest Hemorrhage of the Mouth and Gums.*—Dr. Q. C. Smith writes to *Pacific Med. and Surg. Journal* as follows:

Take fine flour, a sufficient quantity, and work into it balsam of fir to the consistence of thin smooth dough. Then take equal parts of powdered kino and catechu, a sufficient quantity and work it into the dough until it is quite stiff. Roll into masses as large as convenient for introducing into the mouth, and use as many as are necessary to pack the mouth tightly when the patient's mouth is closed, so that every part from which hemorrhage issues may be closely compressed by the medicated mass, to get the mechanical as well as chemical effect of the remedy. Renew the application as often as the mass becomes softened by the saliva so as to render it ineffectual.

We have used this remedy in the way just described in a number of cases, with complete success, after persulphate and pernitrate of iron, and other more or less vaunted styptics had failed to serve any good purposes.

---

"*Nothing New Under the Sun.*—Doctor Mordtmann, of Constantinople, publishes, in the *Gazette Medicale de l'Orient*, some curious details he had discovered in some old Oriental chronicles tending to show that the Siamese twins, as well as the sisters Milly and Christine, had prototypes in former times. According to these Byzantine chronicles, there came from Armenia to Constantinople in the year 744, a monster, consisting of two children born of one mother, These children were attached to one another at the epigastrium, so that they faced each other, the other parts of their bodies being regularly formed. During their sojourn in the Byzantine capital numbers flocked to see this monstrosity; but as the twins were superstitiously regarded by the ecclesiastical authorities as being of bad augury, they were expelled the city, to return again when a comparatively enlightened emperor ascended the throne of the Cæsars. One of these twins died, and one of the most skillful physicians endeavored to divide the survivor from the corpse at the point of juncture, in the hope of saving his life, The operation, however, only served to prolong its duration for three days.—*The London Medical Record.*

---

*Lancing the Gums.*—Dr. James Finlayson concludes a paper in the *British Medical Journal* (Sept. 19, 1874) on the alleged dangers of dentition, and the practice of lancing the gums, with the statement that the chief danger of the wholesale use of the gum-lancet lies in its embodying in practice a theoretical view of the ailment, and so tending to close the mind against further inquiry into the diagnosis, etiology and treatment of infantile disorders.

*Directions for Escaping Colds.*—The observations of Raventhal have shown the existence of a central heat-producing power, and an external radiating surface. A rise or fall of temperature is due to a disturbance of the balance existing normally between these two antagonistic areas. An increase of the heat-producing area may produce fever; or this may be due to a diminution of the cooling processes. The reverse of this leads to a lowering of the body temperature, or to a 'chill." The leading article of *British Medical Journal*, April 11, 1874. presents an admirable description of "how colds are caught." As a sequence of these and the above facts the following practical considerations are deduced, which should be written in letters of gold over the door of every house: [*a*] Never wear wet clothes after active muscular exertion has ceased, but change them at once. [*b*] Meet the loss of body heat by warm fluids and dry clothes. [*c*] Avoid long sustained loss of heat which is not met by increased production of heat. [*d*] Increase the tonicity of the cutaneous vessels by cold baths, etc., so as to educate them to contract readily on exposure. [*e*] Avoid warm and debilitating rooms and temperatures. [*f*] Take special care against too great loss of heat when the skin is glowing. [*g*] Prevent the inspiration of cold air by the mouth by breathing through the nose. Old people and children, the anæmic and convalescent, bear cold badly, and hence should carefully observe the above means for escaping colds.—*Detroit Review of Medicine and Surgery.*

